# Use of Geophysical and Remote Sensing Data for Assessment of Aquifer Depletion and Related Land Deformation

**DOI:** 10.1007/s10712-017-9458-7

**Published:** 2018-01-20

**Authors:** Abdullah Othman, Mohamed Sultan, Richard Becker, Saleh Alsefry, Talal Alharbi, Esayas Gebremichael, Hassan Alharbi, Karem Abdelmohsen

**Affiliations:** 1 Department of Geosciences, Western Michigan University, Kalamazoo, MI 49008 USA; 2 Department of Environmental and Health Research, Umm Al-Qura University, Mecca, 21955 Saudi Arabia; 3 Department of Environmental Sciences, University of Toledo, Toledo, OH 43606 USA; 4 Saudi Geological Survey, P.O. Box 54141, Jeddah, 21514 Saudi Arabia; 5 Department of Geology and Geophysics, King Saud University, P.O. Box 89885, Riyadh, 11692 Saudi Arabia

**Keywords:** GRACE, Radar interferometry, Fossil aquifers, Seismicity, Land deformation, Saudi Arabia

## Abstract

An integrated approach [field, Interferometric Synthetic Aperture Radar (InSAR), hydrogeology, geodesy, and spatial analysis] was adopted to identify the nature, intensity, and spatial distribution of deformational features (sinkholes, fissures, differential settling) reported over fossil aquifers in arid lands, their controlling factors, and possible remedies. The Lower Mega Aquifer System (area 2 × 10^6^ km^2^) in central and northern Arabia was used as a test site. Findings suggest that excessive groundwater extraction from the fossil aquifer is the main cause of deformation: (1) deformational features correlated spatially and/or temporally with increased agricultural development and groundwater extraction, and with a decline in water levels and groundwater storage (− 3.7 ± 0.6 km^3^/year); (2) earthquake events (years 1985–2016; magnitude 1–5) are largely (65% of reported earthquakes) shallow (1–5 km) and increased from 1 event/year in the early 1980s (extraction 1 km^3^/year), up to 13 events/year in the 1990s (average annual extraction > 6.4 km^3^). Results indicate that faults played a role in localizing deformation given that deformational sites and InSAR-based high subsidence rates (− 4 to − 15 mm/year) were largely found within, but not outside of, NW–SE-trending grabens bound by the Kahf fault system. Findings from the analysis of Gravity Recovery and Climate Experiment solutions indicate that sustainable extraction could be attained if groundwater extraction was reduced by 3.5–4 km^3^/year. This study provides replicable and cost-effective methodologies for optimum utilization of fossil aquifers and for minimizing deformation associated with their use.

## Introduction

Subsidence is triggered by natural and/or anthropogenic causes by differential sinking of the Earth’s surface due to volume reduction of subsurface materials or presence and collapse of subsurface voids (Ren et al. 1989). Subsidence due to volume reduction, by natural and/or anthropogenic factors, is generally a slow phenomenon occurring over relatively large areas. Subsidence rates were traditionally measured by ground-based geodetic surveying techniques (e.g., precise differential leveling, global positioning systems, terrestrial lidar, extensometry) (Galloway and Burbey 2011). The advent of Interferometric Synthetic Aperture Radar (InSAR) technologies enabled cost-effective contiguous measurements of land deformation over large areas (Gabriel et al. 1989). These technologies have been successfully applied to the Antelope and Santa Clara Valleys in California, the Las Vegas Valley of Nevada (Galloway et al. 1998; Fielding et al. 1998; Galloway and Hoffmann 2007), and the San Luis Valley of Colorado (Reeves et al. 2011).

Recent applications of radar interferometric techniques enabled detection and measurement of slow, large-area land deformations worldwide (Hooper 2006; Kampes 2006), including the San Francisco Bay Area (Bürgmann et al. 2006), central Mexico (Chaussard et al. 2014), and the Nile Delta in Egypt (Becker and Sultan 2009). The correlation of radar-detected subsidence with relevant temporal and spatial observations provides insights into the factors causing the observed subsidence (e.g., Chaussard et al. 2013; Higgins et al. 2014). These factors frequently include groundwater or oil/gas extraction rates, decline in groundwater levels, and the type of rock and sediment. For example, subsidence of up to 40 mm over 35 days in the Lost Hills and Belridge oil fields of the San Joaquin Valley in California was determined to have been caused by excessive oil extraction from depths of around 700 m below the Earth’s surface (Fielding et al. 1998).

Subsidence and other deformational features, such as fissures, sink holes, and earthquakes, have been reported in many of the arid and semi-arid parts of the world, where progressive groundwater extraction projects were conducted. Using InSAR and Global Positioning System (GPS) data, fissures and land subsidence of up to 6 cm/year were detected (1992–1997) over the Las Vegas Valley due to intensive groundwater extraction (93 km^3^/year; Bell et al. 2002). Significant land subsidence was reported from northeast Iran, near the city of Mashhad; InSAR measurements detected subsidence (∼ 28–30 cm/year; period 2003–2005) along the axis of the Mashhad valley and analysis of piezometric records identified extensive extraction of the aquifer system (65 m of water table decline since 1960s) as the cause of subsidence (Motagh et al. 2007). Earth fissures were reported from the Sarir South agricultural project over the Nubian Aquifer in Libya; water levels declined by more than 6 m causing compaction of compressible fine-grained deposits within the aquifer and development of fissures (El Baruni 1994). Using GPS data from two stations (period 2006–2009), subsidence rates of up to 10 cm/year were reported from the Quetta Valley in Pakistan; subsidence was attributed to excessive groundwater extraction to support population growth (1975: 260,000; 2010: 1.2 million) in the Quetta Valley due to migration of refugees from war-torn neighboring Afghanistan (Khan et al. 2013). Groundwater-extraction-related deformation was reported for both confined and unconfined aquifers (Galloway et al. 1998; Poland 1960). The deformation was either uniform over large areas, or differential, causing fissures, damage in buildings and infrastructure (Burbey 2002), as well as vertical and possibly horizontal displacement of land surfaces (Burbey 2001; Galloway and Burbey 2011).

The largest number of subsidence cases in arid and semi-arid lands is reported over fossil aquifers. At present, precipitation and surface water resources in these areas are limited, intensive irrigation programs are being implemented, and groundwater is the sole source for water supply (Maliva and Missimer 2012). Examples of such areas include the Sahara of North Africa and of the Arabian Peninsula. Aquifers within these areas were largely recharged in the wet climatic conditions of the Pleistocene epoch. Currently, they receive only modest local recharge, which does not compensate for the anthropogenic discharge (Sultan et al. 2014). Examples of such systems are the Mega Aquifer System (MAS) in the Arabian Peninsula (e.g., Hoetzl et al. 1978; Jado and Zötl 1984; Sultan, et al. 2014), the Nubian Aquifer System in northeastern Africa (Mohamed et al. 2017), the North Western Sahara Aquifer System in north western Africa, and the Great Artesian Basin in eastern Australia (Taylor et al. 2013). In such areas, and for those fossil aquifers, the excessive and prolonged extraction of non-renewable groundwater would be expected to cause drawdown of the artesian head, high intergranular stresses, compaction of highly compressible clay beds (aquitards), land subsidence, fissuring, reactivation of faults, and if differential vertical displacement occurs, damage to structures and well casings.

**Fig. 1:**
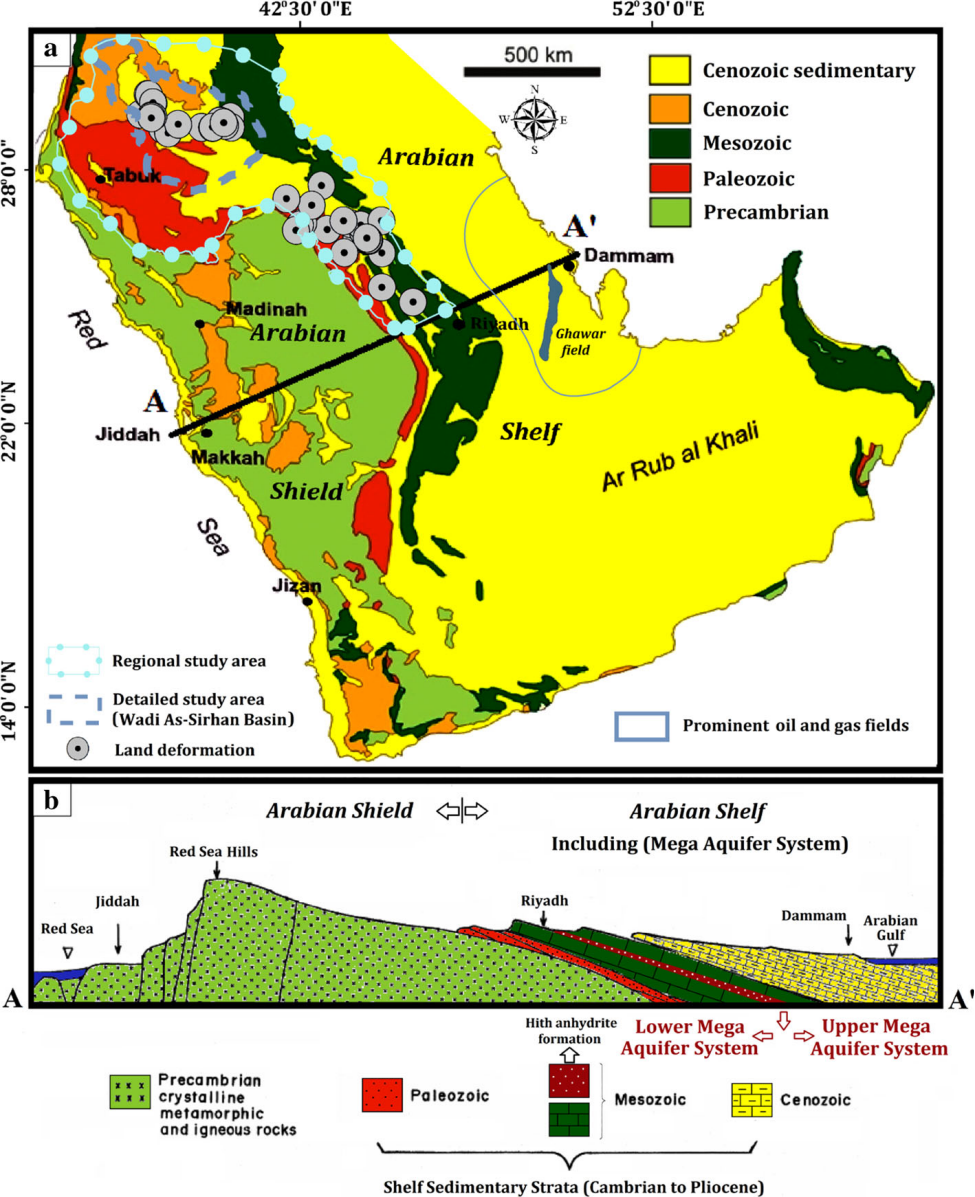
Geologic map and cross section of the Arabian Peninsula adapted after Chapman (1978). **a** Generalized geologic map for the Arabian Peninsula. **b** Cross section along line A–A′ in **a**. **a**, **b** Show the distribution and stratigraphic relations of the Precambrian igneous and metamorphic complex of the Arabian Shield, the overlying Phanerozoic Arabian Shelf rock units, and the multi-layered aquifers of the Phanerozoic Mega Aquifer System (MAS). The location of the regional study is outlined in northern and central Arabia as well as the local study area over Wadi-As-Sirhan

**Fig. 2:**
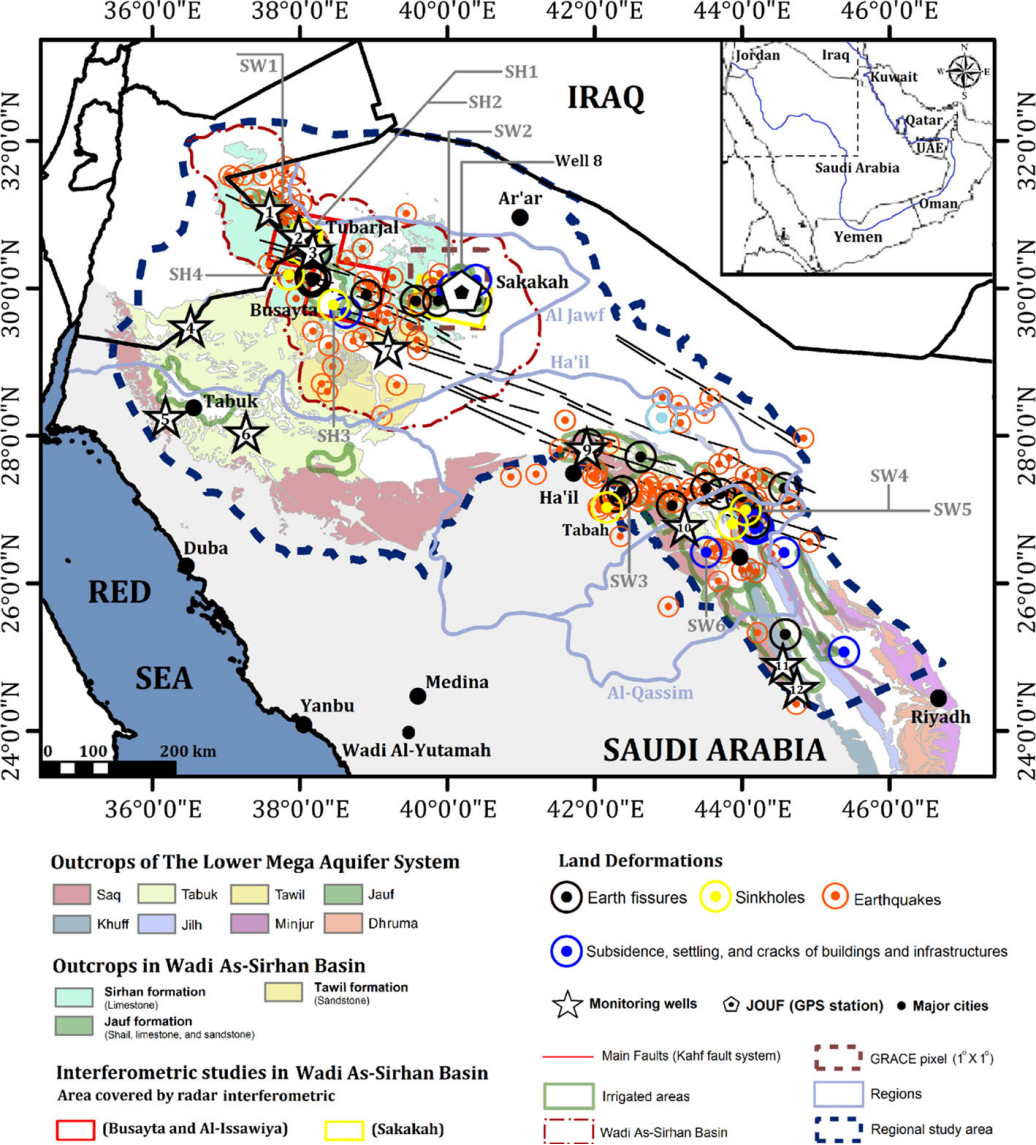
Location map of the regional study area (northern and central Arabia) and the local study area (WASB), showing the distribution of the field-verified land deformation sites, LMAS outcrops, irrigated areas extracted from Landsat thematic mapper data, area covered by the radar interferometric study within the WASB, investigated GRACE pixels, earthquake epicenters from 1982 to 2014 provided by the National Center for Earthquakes and Volcanoes (NCEV) and the JOUF GPS station. Inset showing the distribution of the Mega Aquifer System (MAS) in the Arabian Peninsula

In this paper, we conduct an integrated investigation on one of the largest fossil aquifer systems in the arid world, the MAS of the Arabian Peninsula, review similar settings elsewhere, and compare our findings from the MAS to those reported from similar areas worldwide. The MAS (area ~ 2.3 × 10^6^ km^2^) extends across the Arabian Shelf of the Arabian Peninsula (Fig. 1a) in the Kingdom of Saudi Arabia, Yemen, Oman, United Arab Emirates, Qatar, Kuwait, and Jordan. The aquifer was targeted for agricultural expansions (Fig. 2), and within the reclaimed areas and their surroundings, one or more deformational features and/or structural damage were reported. These areas include the Tabah village, Wadi Al-Yutamah in the southern Medina city, and Wadi Najran (Fig. 2) (Roobol et al. 1985; Vincent 2008; Amin and Bankher 1997; Bankher and Al-Harthi 1999; Youssef et al. 2014).

In the mid-nineteen eighties, the Saudi government embarked on an aggressive agricultural development program in central Arabia (Al-Qassim and Ha’il regions; Fig. 2), which led to an increase in groundwater extraction (1.9 × 10^9^ m^3^/year in 1984 to 4.4 × 10^9^ m^3^/year in 2004) and cultivated lands (from 213 × 10^3^ hectares in 1984 to 316 × 10^3^ hectares in 2004). Similarly extensive agricultural projects were employed in northern Arabia (Wadi As-Sirhan in the Al Jawf region; Fig. 2), where the groundwater extraction increased from 0.095 × 10^9^ in 1984 to 2 × 10^9^ m^3^/year in 2004 and the cultivated areas increased from 14 × 10^3^ hectare to 164 × 10^3^ hectare (MOAW 1984, 2004; Abunayyan and BRGM 2008).

Our integrated study includes both a regional study over the MAS in central and northern Arabia, and a local study over the Wadi As-Sirhan Basin (WASB) (Fig. 1a). The regional study encompassed field investigations to examine land deformational features, and spatial correlation of these features with relevant datasets in search of causal effects. Findings from the regional study were then further investigated and refined with additional datasets (radar interferometric studies and GPS) over the local study area (WASB).

## Geologic, Hydrogeologic, and Climatic Setting of the Investigated Area

The basement complex of the Arabian Shield crops out along the margins of the Red Sea, forming the mountain chain of the Red Sea Hills (Fig. 1). Successions of Phanerozoic sedimentary formations overlie unconformably the basement complex, and dip gently to the east, reaching thicknesses of up to 10 km near the Arabian Gulf (Margat 2007; Powers et al. 1966; Lloyd and Pim 1990; Konert et al. 2001). The outcrops at the foothills of the Red Sea Mountains decrease in age eastward toward the Gulf, with the oldest being of the Cambrian age and the youngest from the Quaternary period (Fig. 1b). The uplift associated with the Red Sea opening exposed the basement complex and the overlying Phanerozoic cover, providing opportunities to recharge aquifers within the Phanerozoic formations where they cropped out. The opening of the Red Sea reactivated many of the older NW-trending sinistral Najd fault systems that cross-cut the Arabian Shield in outcrop and at depth under the sedimentary cover throughout the entire Peninsula (Fairer 1983). The reactivation of the faults was dip-slip in response to the extensional events associated with rifting during the Middle to Late Cenozoic Era (Kellogg and Reynolds 1983; Giannerini et al. 1988). These faults are mapped as the Kahf fault system in Fig. 2. Similar observations pertaining to dip-slip movement on earlier Najd faults were reported in the Eastern Desert of Egypt (Sultan et al. 2011).

Collectively, the limestone and sandstone aquifers within the Phanerozoic sections form one of the largest multi-layered aquifers worldwide (Fig. 1). The MAS was largely recharged under previous wet climatic conditions, yet at present it is still receiving a modest local modern recharge, especially in southwest Arabia where precipitation rates of up to 800 mm/year were reported in the Jazan region (Alharbi et al. 2014). Groundwater Cl-36 dating from the Empty Quarter yielded ages of up to one million years (Sultan et al. 2016). Those previous wet conditions contrast with the current arid climatic conditions of an average annual rainfall of 100 mm/year and extremely limited surface water resources over most of the Arabian Peninsula (Barthélemy et al. 2007).

The main aquifers within the MAS are (Fig. 1): (1) Paleozoic sandstone accumulations; (2) Mesozoic marine carbonates; and (3) Cenozoic carbonate formations of Paleogene age and Neogene sedimentary and volcanic formations (Wagner 2011). The MAS is divided into two main groups separated by the Hith anhydrite formation (Fig. 1). The first is the Upper Mega Aquifer System (UMAS), which includes Biyadh, Wasia, Aruma, Umm Er Radhuma, Rus, and Dammam formations. The second is the Lower Mega Aquifer System (LMAS), which includes the Saq, Wajid, Tabuk, Tawil, Minjur, and Dhruma formations. The areas occupied by the UMAS and LMAS are 180 × 10^4^ and 56.3 × 10^4^ km^2^, respectively.

### Wadi As-Sirhan Basin (WASB)

**Fig. 3:**
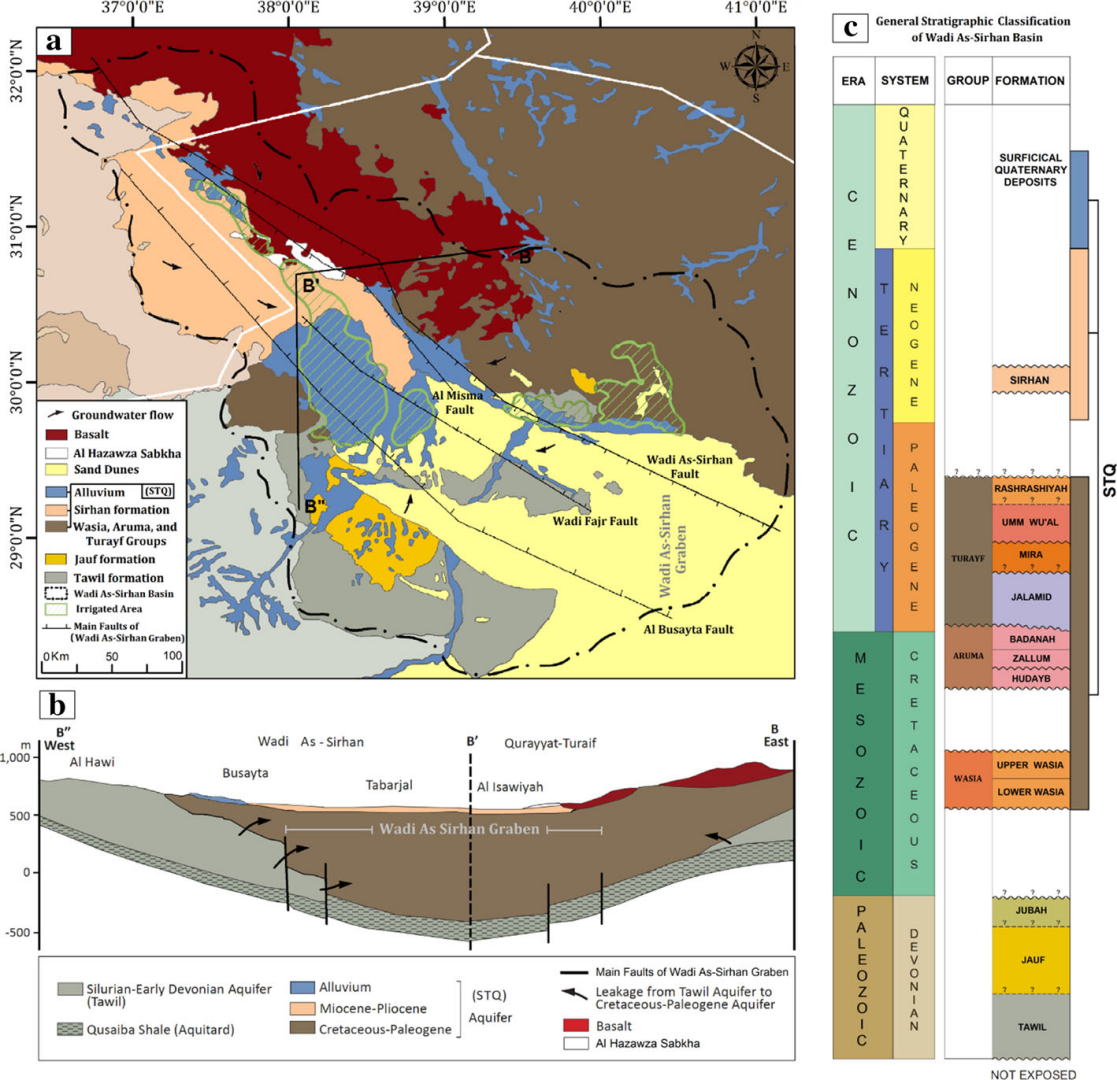
Geologic map, cross section, and stratigraphy of the WASB. **a** Geologic map for the WASB and surrounding areas, showing the distribution of the northwest–southeast-trending Wadi As-Sirhan graben adapted from Wallace et al. (2000). **b** Cross section along line B–B′–B″ in **a** showing the thickness of the lithologic units and aquifers, and groundwater flow directions (modified after UN-ESCWA and BGR 2013). **c** General stratigraphic classification of the WASB adapted from Halawani (2001)

The UMAS is absent in northern and northwestern Arabia, the area including the WASB. In this area, the LMAS is comprised of the Saq (the primary aquifer, yet it is deep and untapped) and the overlying Tabuk, Tawil, Jauf, Jubah, and STQ (Secondary, Tertiary, and Quaternary complex) as local productive aquifers (Fig. 3). The WASB is formed of Devonian sandstone (Tawil formation), Paleogene sedimentary rocks (Turayf group), Neogene (Sirhan formation) and Quaternary basalt, alluvial deposits, gravels, Khabras (silt and clay cemented by evaporite minerals), and sabkhas within depressions in the northwestern section of the study area (Wallace et al. 2000) (Fig. 3). The principal structure in this area is a graben complex with a vertical displacement of up to 1500 m (UN-ESCWA and BGR, 2013) and is bounded by major faults from the east and west, which probably formed by tensional forces associated with Red Sea rifting (Meissner et al. 1990). The faults act as conduits for groundwater discharge, as evidenced by the presence of mud flats, salt lakes, and sabkha (e.g., Al Hazawza Sabkha) within ground depressions and proximal to the faults (ACSAD 1983; UN-ESCWA 1990).

Devonian productive aquifers that are part of the LMAS are found within depressions. Those include the Tawil (Central WASB), Jauf, Jubah (southern WASB), and Cretaceous to Quaternary STQ complex aquifers (Secondary–Tertiary–Quaternary) in the central to northern WASB. Since the 1990s, most of the production has been from the confined parts of the Tawil aquifer in the WASB (Abunayyan and BRGM 2008).

## Methodology

The regional study encompassed: (1) field investigations to examine land deformation features and to generate a “land deformation database”; and (2) spatial analysis (in a GIS environment) of field data with relevant data sets in search of potential regional relationships and causal effects. The latter data sets include lithologic and structural maps, seismic records (Al-Amri 1998), Terrestrial Water Storage (TWS) and Ground Water Storage (GWS) from Gravity Recovery and Climate Experiment (GRACE) solutions (Tapley et al. 2004), GPS measurements, water levels, and land-use maps (Fig. 2). Similar approaches that integrate GRACE and InSAR-derived observations were used to assess groundwater depletion in Central Mexico and to support water resources management in the area (Castellazzi et al. 2016a, b). Findings from the regional study were then further investigated and refined over the local study area (Wadi As-Sirhan), where observations from the aforementioned datasets were integrated with those extracted from radar interferometric measurements (Fig. 2). Two areas within the WASB were targeted for radar interferometric studies; the first was Busayta and Al-Issawiyam, and the second was Sakakah (Fig. 2). These two areas are hereafter referred to as the Busayta and Sakakah regions. These two areas were selected because they had the largest coverage of ENVISAT radar imagery and are proximal to the GPS Sakakah station.

### Field Observations

Over the past decade, there has been an increase in reported incidences of land deformation events that resulted in loss of life and property, especially from the northern and central parts of Arabia. Field trips were conducted in 2004, 2010, and 2015 to investigate: (1) deformation events reported from central (Al-Qassim and Ha’il regions) and northern (Al Jawf region) Arabia; (2) deformational features (sinkholes) extracted from temporal high-resolution satellite images; and (3) locations where displacement were extracted from InSAR data. Throughout the fieldwork, one or more of the following deformational styles were observed and documented in 37 locations: subsidence, sinkholes, earth fissures, and cracks and damages to buildings, structures, and infrastructure due to differential settlement of their foundations. Specifically, six large sinkholes (diameter 15–40 m), five smaller sinkholes (diameter 1–3 m), earth fissures at 14 sites (fissure widths 0.18–2 m), and cracks in roads and buildings in 12 locations were documented. In the search for causal effects, we collected ancillary field data, including proximity to agricultural developments and urban areas, agricultural practices (e.g., groundwater extraction, excessive watering), presence or absence of sewage systems, temporal variations in groundwater levels, soil and bedrock type, and distribution of fractures/fault systems. Of these features, 26 were located in and around agricultural lands, while the remaining 11 locations were in urban areas proximal to the agricultural lands.

### Gravity Recovery and Climate Experiment (GRACE)

GRACE is a joint satellite mission (launched in March 2002) between the National Aeronautics and Space Administration (NASA) in the USA and the German Aerospace Center (DLR) to measure temporal variations in the Earth’s gravity field (Tapley et al. 2004) that are related to TWS spatiotemporal variations (Wahr et al. 1998; Wouters et al. 2014; Ahmed et al. 2014, 2016; Mohamed et al. 2017; Sultan et al. 2013, 2014). TWS refers to the vertically integrated measurement of water storage that includes groundwater, soil moisture, surface water, snow water, and vegetation water (Strassberg et al. 2007).

The recently released (release 05; RL05), high resolution (grid size 0.5° × 0.5°), monthly mass concentrations (mascon) solutions (Save et al. 2016) spanning the investigated period (April 2002 to June 2016) were analyzed to estimate the temporal variations in TWS over the lower MAS (regional study) and over the WASB (local study). Data were obtained from the Center for Space Research (CSR) at the University of Texas^1^(Save et al. 2016) and were used without any prior processing, filtering, or empirical scaling factors (Save et al. 2016).

The secular trend in GRACE-derived TWS data was extracted by fitting a linear regression applied to each TWS time series, and the error in the trend was estimated using procedures described in Scanlon et al. (2016). The change in groundwater storage was estimated using the following equation:

1 $$\Delta {\text{GWS}} = \Delta {\text{TWS}} \,{-} \,\Delta {\text{SMS}}$$

where ΔGWS and ΔSMS represent the change in groundwater and soil moisture storage, respectively. The soil moisture was extracted from the Global Land Data Assimilation System (GLDAS) model, a NASA-developed land surface modeling system that simulates climatic and hydrologic variables (Rodell et al. 2004). The soil moisture time series over the investigated areas was calculated by averaging the soil moisture estimates from four GLDAS model versions: Variable Infiltration Capacity (VIC), Community Land Model (CLM), Noah, and MOSAIC (Rodell et al. 2004; Dai et al. 2003). The errors ($\sigma_{\text{SM}}$) were estimated from the standard deviation of the trends that were computed from the four GLDAS simulations. The trend error in GWS ($\sigma_{\text{GWS}}$) was calculated using standard error propagation equations:

2 $$\sigma_{\text{GWS}} = \sqrt {(\sigma_{\text{TWS}} )^{2} + \left( {\sigma_{\text{SM}} } \right)^{2} }$$

### Radar Interferometric Studies

Twenty-nine descending synthetic aperture radar (SAR) scenes (Table 1) were acquired from the European Remote Sensing ENVISAT satellite. Twenty-one of those scenes were acquired over the Tabarjal and Busayta area (tracks 221, 351 and 493) and the remaining eight scenes over the Sakakah and Dumat Aljandal area (track 178). The investigated period for Busayta was from August 2003 to January 2012, and for the Sakakah area it was from December 2003 to July 2008. The spatial baseline ranged from 17 to 835 m with respect to the master scenes (acquisition date of master scenes: May 17, 2004, August 01, 2007, and March 21, 2011) for the Busaytah area, and from 38 to 750 m in Sakakah area (acquisition date of master scene: November 11, 2007). The maximum temporal baseline was 1434 days for both areas. For the majority of the investigated areas, the density of permanent scatters ranged from 10 to 20 persistent scatterers/km^2^ with a coherence threshold value of 0.4 and an amplitude dispersion value of 0.4.

**Table 1:** Envisat scenes used in Persistent Scatterers study

Area	Satellite	Acquisition date	Perpendicular baseline (m)	Temporal baseline (days)	Track
Tabarjal	ENVISAT	20030811	− 646	− 279	493
Tabarjal	ENVISAT	20031124	− 262	− 174	493
Tabarjal	ENVISAT	20040202	835	− 104	493
Tabarjal	ENVISAT	20040308	338	− 70	493
Tabarjal	ENVISAT	20040517	0	0	493
Tabarjal	ENVISAT	20040621	− 17	35	493
Tabarjal	ENVISAT	20071203	409	1295	493
Tabarjal	ENVISAT	20080421	420	1434	493
Busayta	ENVISAT	20031210	− 421	− 1101	221
Busayta	ENVISAT	20040602	660	− 927	221
Busayta	ENVISAT	20060329	− 756	− 350	221
Busayta	ENVISAT	20070314	404	− 140	221
Busayta	ENVISAT	20070801	0	0	221
Busayta	ENVISAT	20090527	233	664	221
Busayta	ENVISAT	20101121	651	− 120	351
Busayta	ENVISAT	20101221	588	− 90	351
Busayta	ENVISAT	20110321	0	0	351
Busayta	ENVISAT	20110520	316	60	351
Busayta	ENVISAT	20110619	− 190	90	351
Busayta	ENVISAT	20111017	− 394	210	351
Busayta	ENVISAT	20120115	− 705	300	351
Sakakah	ENVISAT	20031207	− 471	− 1410	178
Sakakah	ENVISAT	20040425	− 101	− 1295	178
Sakakah	ENVISAT	20040530	379	− 1260	178
Sakakah	ENVISAT	20040704	− 691	− 1225	178
Sakakah	ENVISAT	20040808	− 284	− 1190	178
Sakakah	ENVISAT	20060813	750	− 455	178
Sakakah	ENVISAT	20071111	0	0	178
Sakakah	ENVISAT	20080713	38	275	178

Temporal and perpendicular baselines are shown relative to the scene acquired on May 17, 2004, August 01, 2007, and March 21, 2011 for area d (Tabarjal and Busayta) and November 11, 2007 for area e (Sakakah)

We applied Persistent Scatterer Interferometry (PSI) techniques (Hooper et al. 2004, 2007) to investigate the spatial variations in subsidence rates across the WASB in two sites (Tabarjal, Busayta, Sakakah and Dumat Aljandal areas) (Figs. 2, 10). The PSI method restricts the phase unwrapping and analysis to coherent pixels, which contain individual scatterers and remain stable over the investigated time period. In the study area, these scatterers represent buildings, outcrops, rocks, dwellings, utility poles, and well foundations that were identified using the Stanford Method for Persistent Scatterers (StaMPS) algorithm (Hooper et al. 2007, 2012). Precise orbit information was obtained from Delft Institute for Earth-Oriented Space Research (Scharroo and Visser 1998); the interferometric processing was performed using the Delft object-oriented radar interferometric software (DORIS) (Kampes et al. 2003), the Repeat Orbit Interferometry Package (ROI_PAC), and the StaMPS algorithm (Hooper et al. 2004).

### Global Positioning System (GPS) Station

Three-dimensional global positioning system data from the JOUF Continuously Operating Reference Station (CORS)^2^ of the Saudi Ministry of Municipal and Rural Affairs (MOMRA) Real-Time Network (MRTN) network station (Sakakah city; lat 29.962°N; long 40.199°E) was used to detect and quantify the horizontal motion of the Arabian plate as well as the vertical land deformation in the study area. The CORS network provides detailed and accurate real-time position outputs without requiring a reference base station, unlike traditional Global Navigation Satellite System (GNSS) surveying observation techniques (Al Omar et al. 2015). The JOUF station data were analyzed using the Orbit Analysis Simulation Software (GIPSY) developed by the Jet Propulsion Laboratory (JPL) at NASA (Zumberge et al. 1997) to extract the deformation rate (averaged for every minute) and the temporal pattern in 2015 for the southern part of WASB.

### Landsat Images

Temporal Landsat (5, 7, and 8) Thematic Mapper (TM) imagery (path 172 and row 39) acquired over WASB (acquisition dates of February 1987, 1991, and 2000, March 2003, January 2014, and February 2017) were processed to track the development of agricultural activities throughout the past three decades (1987–2017). The Landsat 5, 7, and 8 missions were launched in March 1984, April 1999, and February 2013, respectively, as collaborative efforts between the National Aeronautics and Space Administration (NASA) and the United States Geological Survey (USGS). Temporal variations in the areas occupied by agricultural developments were assessed using the Normalized Difference Vegetation Index (NDVI) images. Using the reflection from the red and near infrared band, an NDVI value was extracted using standard calculations (Rouse et al. 1974):

3 $${\text{NDVI}} = \left({\text{near}}\,{\text{infrared}}{-}{\text{red}} \right)/\left( {{\text{near}}\,{\text{infrared}} + {\text{red}}} \right)$$

The higher the NDVI values, the greater the density of vegetation, and vice versa. The areas occupied by cultivated land were extracted from the Landsat imagery using a threshold NDVI value of 0.3; the threshold value was validated by visual comparisons with Google Earth images.

### Arabian Peninsula Seismographic Database

Until 2003, there were three independent analog seismic telemetry networks with 75 seismograph stations in Saudi Arabia: (1) the King Saud University (KSU) network that was established in 1985; (2) the King Abdulaziz City for Science and Technology (KACST) network that was established in 1993 (Al-Amri 1998); and (3) the Saudi Geological Survey (SGS).

Commencing in 2005, all three networks were integrated under the National Centre for Earthquakes and Volcanoes (NCEV) as part of the SGS. This network monitors earthquakes across the Arabian Peninsula and the Middle East (Al-Amri et al. 2008). Archival seismic data from 1965 onwards, compiled at the International Seismological Center (ISC) and from the Preliminary Determination of Epicenters (PDE) (Ambraseys 1988), were acquired from King Saud University (Al-Amri 1998) and from the NCEV. A total of 115 records were provided for earthquakes that occurred in northern and central Arabia throughout the period 1980–2016. Each record included epicenter location, magnitude, and depth for a subset (65 records) of the data.

### Spatial Database in GIS

The adopted approach for processing spatial datasets in a GIS environment entailed: (1) compiling relevant spatial (e.g., rock and soil types, structures, etc.) and temporal (e.g., groundwater extraction, landuse/landcover change, distribution and magnitude of earthquakes) datasets; (2) constructing a GIS database to host, organize, manage, and analyze the data sets; and (3) conducting spatial correlations of the reported land deformation locations and extracted InSAR deformation with the generated digital products (e.g., soil types, groundwater extraction, TWS, GWS) in search for possible causal relationships.

The generated database incorporates co-registered digital mosaics with a unified projection (type: UTM Zone 37, datum: WGS-1984) including: (1) regional (scale 1:4,000,000) and detailed (scale 1:250,000) geologic maps; (2) digital elevation models (DEM; spatial resolution 30 m), generated from Level 1A Advanced Spaceborne Thermal Emission and Reflection Radiometer (ASTER) scenes; (3) Landsat 5, 7, and 8 TM scenes and NDVI products (spatial resolution: 30 to 60 m); (4) distribution of fractures/fault systems extracted from geologic maps and subsurface data; (5) distribution of the reported land deformation locations; (6) spatial and temporal distribution of earthquake epicenters; (7) water well locations; (8) temporal groundwater extraction volumes and water levels; (9) distribution of the irrigated areas; (10) GRACE-derived TWS and GWS trend (mm/year) maps over the Arabian Peninsula; (11) temporal three-dimensional GPS measurements; and (12) PSI results over Busayta and Sakakah areas in WASB.

## Data Analysis: Field, GRACE, Seismicity, and GPS

This section details the spatial and temporal correlations for the ancillary datasets in support of the hypothesis that excessive extraction from the LMAS in central and northern Arabia was largely responsible for the observed land deformation, the detected subsidence from PSI analysis, and the recent seismic activity.

### Regional Study

**Fig. 4:**
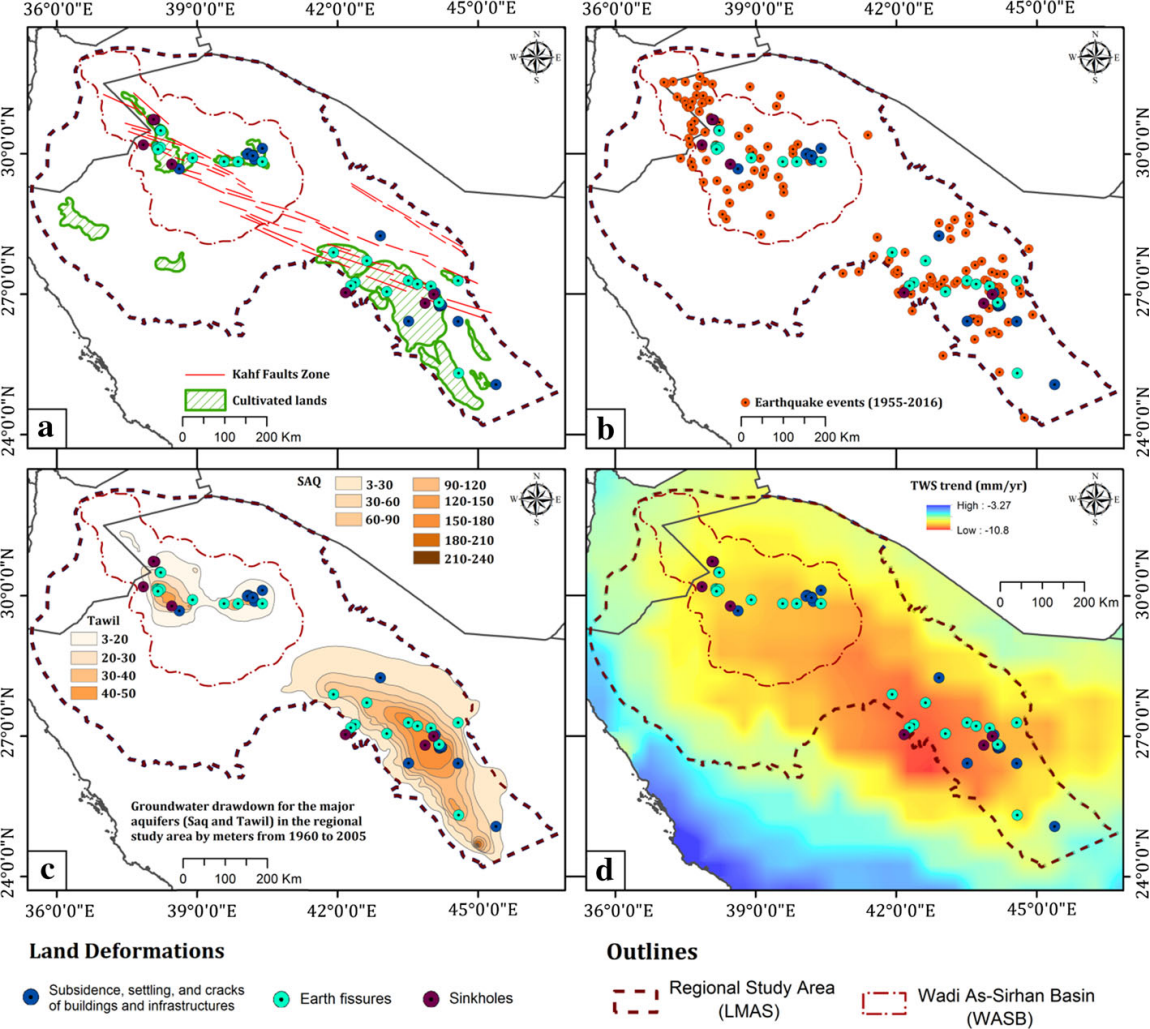
Distribution of land deformation over LMAS in relation to: **a** the distribution of cultivated land and Kahf fault traces adapted from Khalil (2016), **b** earthquake epicenters reported in years (1982–2014) provided by Seismographic Network Datasets (Al-Amri 1998), **c** groundwater drawdown for the major aquifers (Saq and Tawil) in the regional study area (LMAS) modified after Abunayyan and BRGM (2008) and (d) TWS secular trend (mm/year) derived from GRACE 1° × 1° Mascon solutions acquired (04/2002–03/2016) over the regional study area (LMAS)

From field investigations over the study area, it was determined that most (70%) of the identified land deformation sites were located in and around cultivated lands (Figs. 2, 4a), and a few sites (24%) were identified within urban areas (small village) that are proximal (< 20 km) to cultivated areas. The majority of the fissures (14 sites) trended in a northwest–southeast direction and were proximal and sub-parallel to the widely distributed northwest-trending Kahf fault system in central and northern Arabia, including the WASB study area (Figs. 2, 4a).

The distribution of the identified deformation sites spatially correlated with the distribution of seismically active areas (Fig. 4b), areas witnessing excessive extraction (Fig. 4c), and areas showing TWS depletion (Fig. 4d). The majority (90%) of the reported earthquakes in northern and central Arabia cluster within, or proximal (5–10 km) to, the area occupied by the LMAS (Fig. 2). Earthquake epicenters are close to the cultivated lands (Fig. 4a, c), their magnitude is small (1–5M), and their depths are shallow. Out of the 49 earthquakes whose epicenter depths were estimated, all but three had depths of 10 km or less, two at 12 km, and 1 at 14 km.

**Fig. 5:**
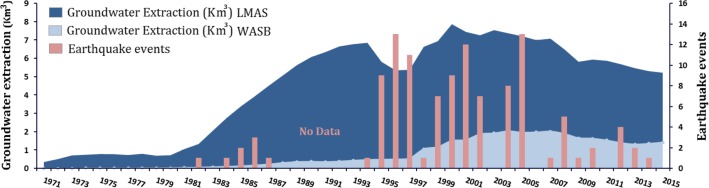
Comparison between the temporal variations in groundwater extraction and the frequency of earthquake (> 1 Magnitude) events (1982–2014) over the regional study area (LMAS) and Wadi As-Sirhan Basin (WASB). Data provided by the Water Resources Development Department in the Ministry of Environment, Water and Agriculture

Figure 5 indicates that the onset of seismic activity in the study area began in the early eighties, which was the same time that the intensive groundwater extraction program was launched, and peaked in the 1990s and early 2000s, the period that witnessed the highest groundwater extraction rates. Until 1980, extraction in the study area was minimal (< 1 km^3^/year) and continued to increase until the 1990s and early 2000s, after which it leveled off to around 7–8 km^3^/year by 2007, followed by a decline to approximately 5.5 km^3^/year by 2015 (Fig. 5). The reported groundwater extraction quantities in Fig. 5 represent the cumulative extraction amounts from Al-Qassim, Ha’il and Al Jawf regions (Fig. 2).

**Fig. 6:**
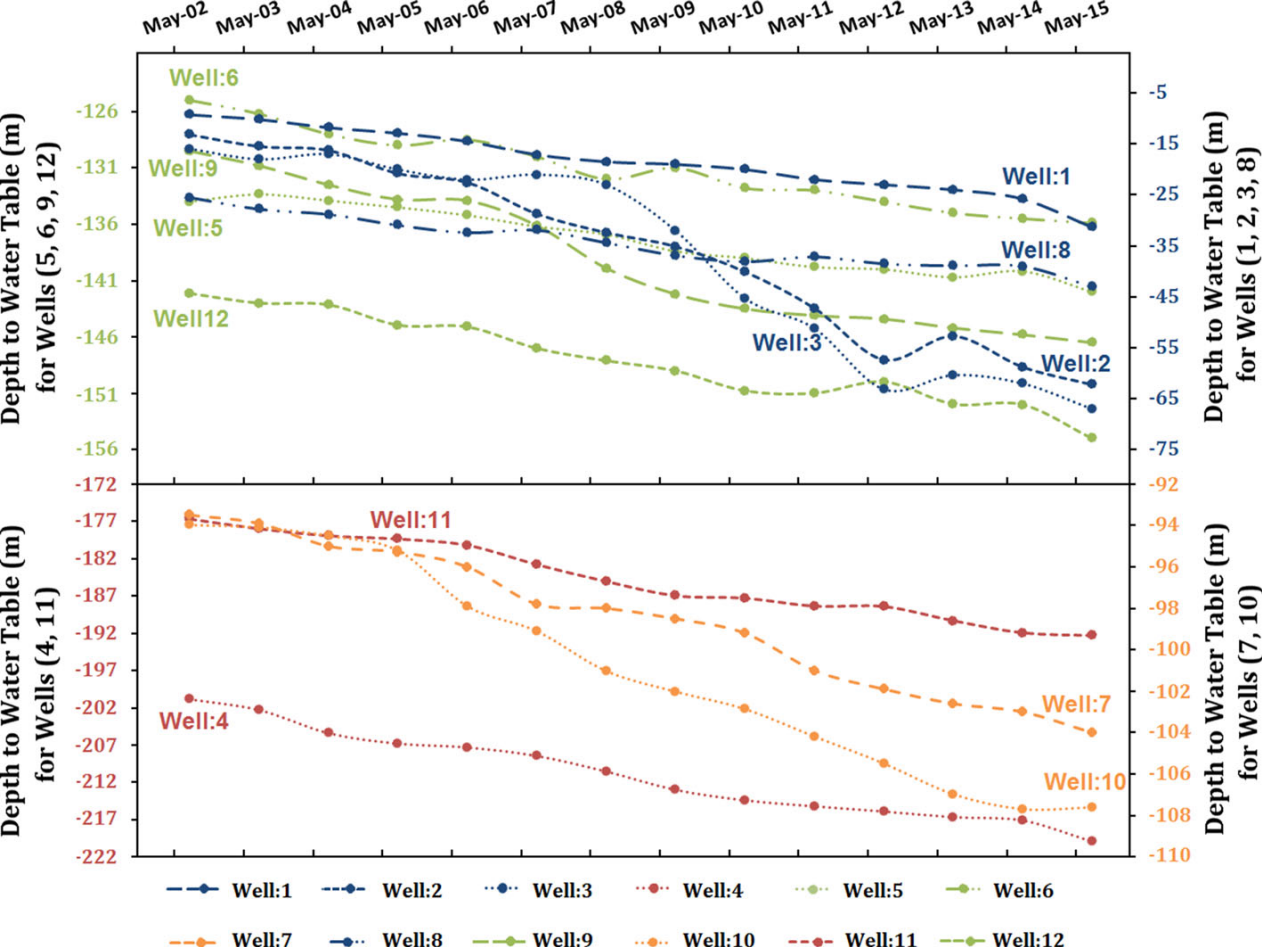
Water levels (2002–2015) from twelve monitoring wells drilled in the recharge areas of the LMAS provided by the Water Resources Development Department in the Ministry of Environment, Water and Agriculture

Between the field campaigns in 2004 and 2010, a significant increase in cultivated land was observed, shallow wells (depth range < 75 m) dried up (e.g., Figure 2: SW1, SW2, and SW4), sinkholes (e.g., Fig. 2: SH1, SH2, and SH3) increased in size (diameter increased by more than 5 m) and the shallow water in the sinkholes dried up. The decline in groundwater levels observed during our field trips was consistent with observations from monitoring wells provided by the Water Resources Development Department in the Ministry of Environment, Water and Agriculture in Saudi Arabia.^3^ The selected monitoring wells were located over LMAS outcrops and were distant (20–100 km) from cultivated lands, where excessive groundwater extraction occurs. The excessive extraction was unsustainable, as evidenced by the decline in water levels. A 50 m drop in water levels was observed in the WASB depression (Figs. 2, 6: wells 2 and 3), 18 m in the northern sections of the regional study area (Figs. 2, 6: wells 1, 4 and 8), and 9 to 17 m in the central and southern sections (Figs. 2, 6: wells 9, 10, 11, and 12).

**Fig. 7:**
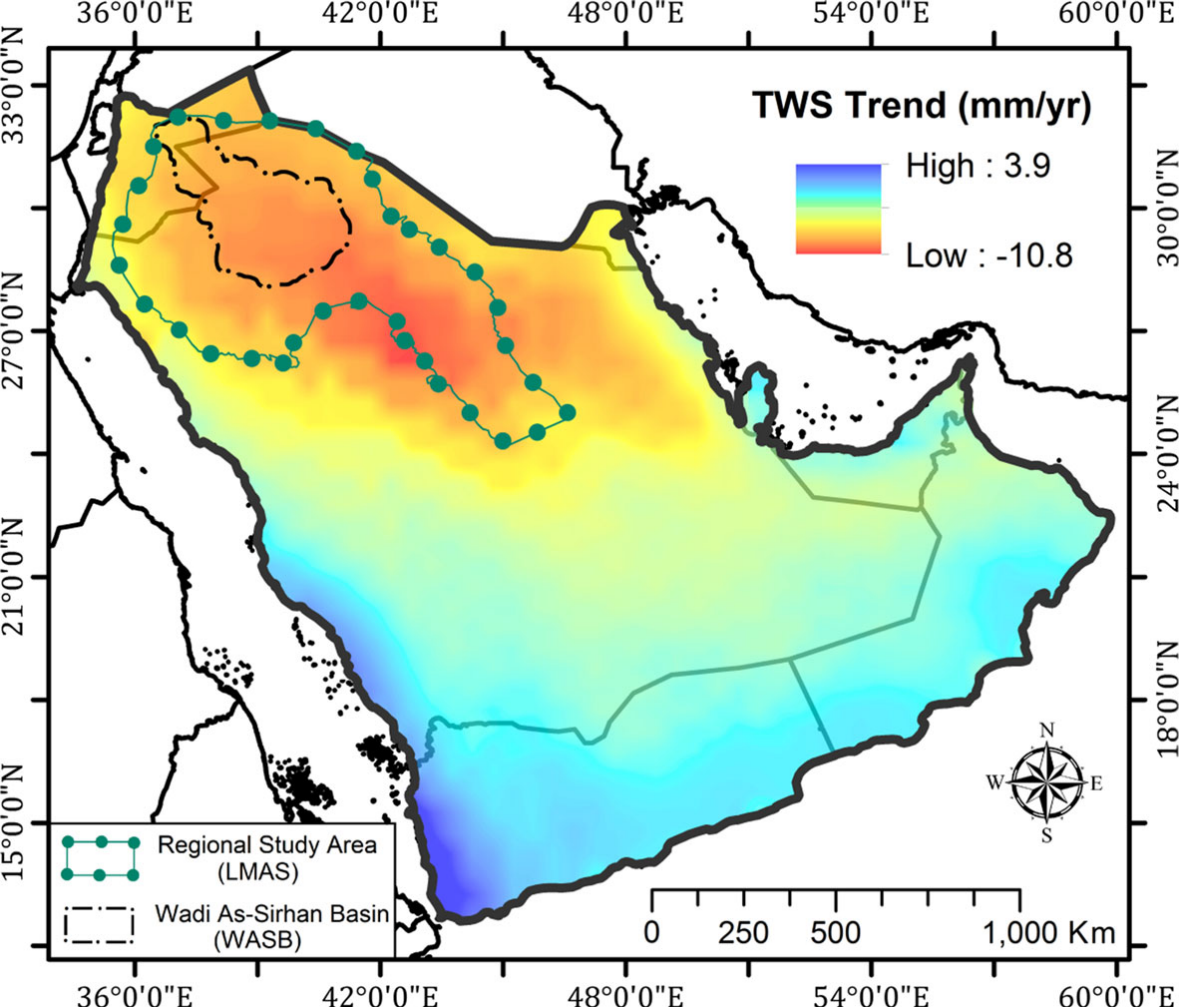
Secular trend (mm/year) for GRACE-derived TWS for the Arabian Peninsula extracted from temporal (04/2002–03/2016) CSR 1° × 1° Mascon solutions showing high TWS depletion rates over the study areas. The outlines of the two areas used for deriving time series for Fig. 8 are also shown

**Fig. 8:**
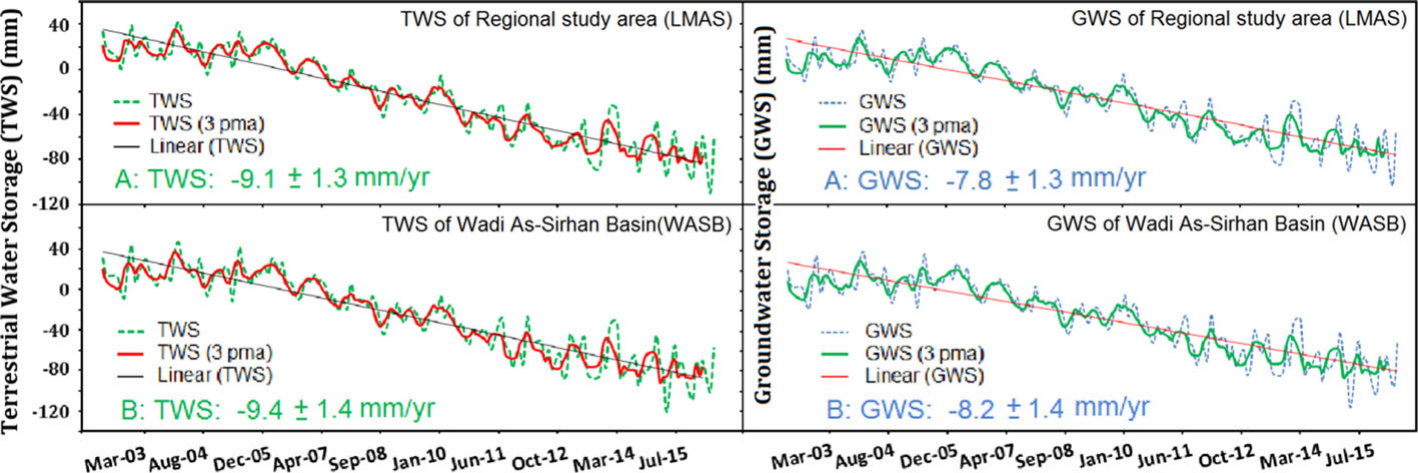
Time series and secular trends for TWS and GWS over the regional study area (LMAS) and Wadi As-Sirhan Basin (WASB) outlined in Fig. 7

The excessive extraction and unsustainable utilization of the LMAS was reflected in both the GRACE solutions and the GPS measurements. Depletions in GRACE-derived TWS and GWS over LMAS and WASB were observed. The spatial distribution of the secular trends in GRACE-derived TWS data over the Arabian Peninsula is shown in Fig. 7. Positive trends indicate an increase in TWS with time and negative trends indicate the opposite. Figure 7 depicts that the areas occupied by the LMAS and the WASB were experiencing significant negative TWS trends (shades of yellow, orange, and red), compared to areas to the south that ranged from lower depletion (shades of cyan) to near-steady conditions (shades of blue) along the Red Sea coastal plain. Figure 8 shows the temporal variations in GRACE-derived TWS, and the secular trend over the LMAS (− 9.1 ± 1.3 mm/year; − 4.6 ± 0.5 km^3^/year) and the WASB (− 9.4 ± 1.4 mm/year; − 1.0 ± 0.1 km^3^/year). Using GRACE-derived TWS data combined with GLDAS-derived soil moisture data applied to Eq. 1, GWS time series for each of the LMAS and the WASB were extracted (Fig. 8; Table 2).

**Table 2:** Partitioning of Terrestrial Water Storage (TWS) over the regional study area (LMAS) and Wadi As-Sirhan Basin (WASB)

Area	Area	ΔTWS		ΔSMS		ΔGWS	
	km^2^	mm/year	km^3^/year	mm/year	km^3^/year	mm/year	km^3^/year
Regional study area (LMAS)	0.44 × 10^6^	− 9.1 ± 1.3	− 4.6 ± 0.5	− 1.3 ± 0.2	− 0.6 ± 0.1	− 7.8 ± 1.3	− 3.7 ± 0.6
Wadi As-Sirhan Basin (WASB).	0.1 × 10^6^	− 9.4 ± 1.4	− 1.0 ± 0.1	− 1.2 ± 0.2	− 0.13 ± 0.02	− 8.2 ± 1.4	− 0.9 ± 0.1

GRACE observations, LSMS outputs, and field data were used to estimate the partitioning of TWS in SMS, and GWS over areas occupied by the LMAS and WASB

Δ*TWS* change in terrestrial water storage, Δ*SMS* change in soil moisture storage, Δ*GWS* change in groundwater storage

**Fig. 9:**
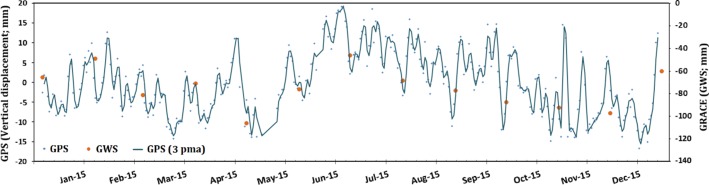
Vertical displacement rate from the JOUF GPS station versus GWS mascon solutions over a single 1° × 1° pixel. Locations of the JOUF station and the GRACE pixel are shown in Fig. 2

The observed TWS and GWS annual cycle were largely controlled by groundwater extraction. Temperatures in summers (June–August) are high (range 28 to 37 °C), compared to winter temperatures (range 5 to 20 °C). Evaporation rates are as high as 28 mm/day in the summers, and as low as 0.4 mm/day in the winters (Alsharhan et al. 2001). As a result, agricultural activities and groundwater extraction increases in the winter season (December–February) and decline in the summers. This is supported by the correspondence between the GRACE solutions and the vertical displacements extracted from the JOUF (January 2015 to December 2015) GPS station (Fig. 9). Excessive extraction in the winter caused water-level declines, and land subsidence and the replenishment in the summer season by groundwater flow, caused a rebound. Given that there was a depletion rate of − 3.7 ± 0.6 km^3^/year in GWS over the regional study area, it could be interpreted that the replenishment does not fully compensate for the extracted groundwater.

### Wadi As-Sirhan Basin

**Fig. 10:**
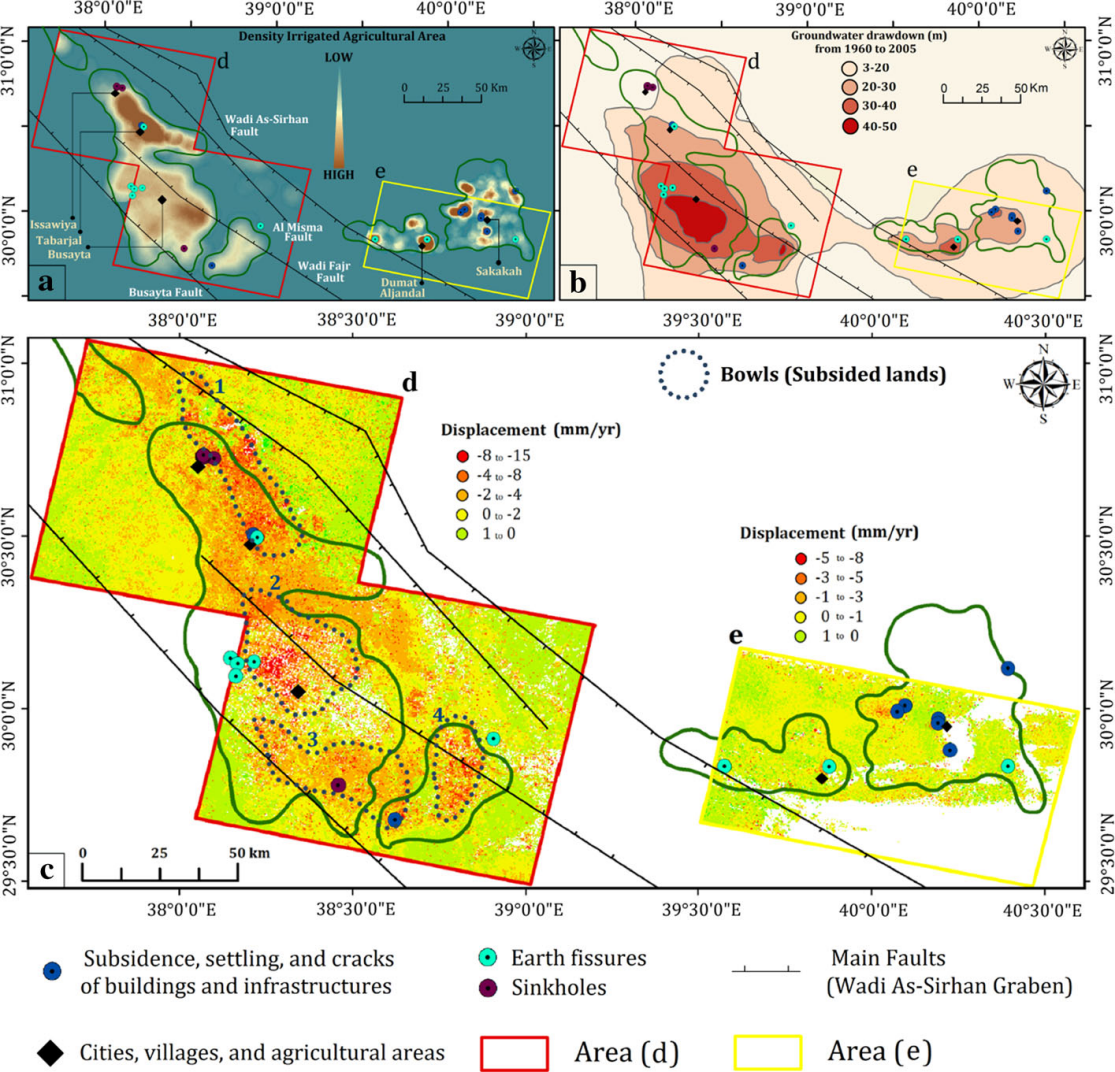
Distribution of land deformation over the Wadi As-Sirhan in relation to: **a** density of irrigated lands, **b** groundwater drawdown (1960–2005) modified after Abunayyan and BRGM, 2008, and **c** PSI results over the Busayta, Tabarjal, and Issawiya areas (area “d” outlined by red polygon) and the Sakakah and Dumat Aljandal region (area “e” outlined by yellow polygon). The distribution of the major faults are also depicted with black lines (modified after Wallace et al. 2000)

The Wadi As-Sirhan area is part of the regional study area and displays correlations between the locations of deformation sites and the distribution of agricultural areas, areas affected by the Kahf faults and seismic activity, areas witnessing drawdown in water levels, and depletions in GRACE-derived TWS and GWS (Figs. 2, 4, 7, 10). To achieve a better understanding of the distribution, nature, and factors controlling the ongoing land deformation in the study area, PSI techniques were applied (Hooper et al. 2004, 2007, 2012) using stacks of ENVISAT ASAR scenes acquired throughout the period 2003 to 2012 over two locations (Busayta and Sakakah) within the Wadi As-Sirhan area (Table 1). Both regions encompass cultivated lands; the Busayta is heavily cultivated (87% of investigated area) and is considered to be the food basket of Saudi Arabia, whereas the Sakakah region has far less cultivated lands (13% of the investigated area). As a result, less groundwater is extracted.

The applied Persistent Scatterer technique enabled successful interferometric applications over a wide range of physiographic and atmospheric conditions, including highly vegetated lands (Becker and Sultan 2009) similar to the ones being investigated in Wadi As-Sirhan.

Figure 10c shows the surface displacement expressed as velocities along the radar line of sight and displays land deformation patterns over the two regions of interest. In this figure, areas moving away from the satellite (subsiding) appear in shades of red and orange, whereas stable features (0–1 mm/year) appear in shades of green and yellow. Figure 10c also shows that subsiding areas (− 2 to − 15 mm/year) cover extensive domains (41% of the InSAR investigated area) in the Busayta region and are located along a NW–SE-trending fault zone that bounds Wadi As-Sirhan graben. Specifically, the subsiding areas within the graben are bound by two major faults: the Wadi As-Sirhan fault in the north and the Al Busayata fault in the south (Fig. 10a, c). Subsiding areas were found within, or proximal to, cultivated areas (NDVI > 0.3) and regions experiencing large drops in groundwater levels (Fig. 10a–c). Generally, subsidence was high (− 2 to − 15 mm/year), yet irregularly distributed within the graben, compared to areas to the east and west of the bounding faults, which showed minimal or a lack of subsidence. The highest subsidence rates were localized within elongated bowls, many of which were proximal to, or bound by, the major faults in the area. A number of the observed fissures were located along the peripheries of the subsiding bowls (Fig. 10c).

For the Sakakah region, the subsiding areas (5% of the InSAR investigated area) and the subsidence rates (− 3 to − 8 mm/year) were small compared to the Busayta region. This is attributed to: (1) smaller cultivated areas (13% of InSAR investigated area) compared to the Busayta region (36% of InSAR investigated area); (2) modest groundwater extraction (0.17 km^3^) and drop in water levels (up to 1.5 m/year) compared to Busayta (groundwater extraction: 2.3 km^3^; drop in water level: up to 4 m/year); and (3) a thinner aquifer (thickness: 260 to 300 m) compared to the Busayta region that lies within a graben with thickness ranging from 600 to 980 m in the center of the depression (Fig. 3b).

## Findings and Implications

This study demonstrated that the land deformation-related features (e.g., sink holes, fissures, subsidence, and earthquakes) lie to the northwest of, and are distant (300–1500 km) from, the prominent oil and gas fields including the Ghawar and surrounding fields (Fig. 1: area outlined by blue polygon) which produce the bulk of the Saudi oil and gas (Fox and Ahlbrandt 2002) suggesting that oil and gas extraction is not the cause of the deformation. However, these land-deformation features are spatially correlated with features associated with agricultural development and groundwater extraction in central and northern Arabia, suggesting a causal effect (Figs. 2, 4a–c, 6a, b). The increase in cultivated land was associated with a decline in water levels and GWS and TWS depletion (Fig. 8). Not only were those correlations observed spatially, but also temporally. For example, earthquake events increased from one event/year in 1980, the year that witnessed the onset of the agricultural development program in central and northern Arabia (extraction: 1 km^3^/year), up to thirteen events/year in the 1990s, the decade that witnessed the largest expansion in groundwater extraction (average annual > 6.4 km^3^) and land reclamation (Fig. 5). The reported earthquakes are shallow (1 to < 10 km) and their magnitude is small (1–5M). These observations support the theory that excessive groundwater extraction was responsible for the observed land deformation.

Pumping from limestone aquifers such as the Aruma and Sirhan could result in the loss of buoyant support to roofs of preexisting cavities, causing collapse, and the development of sinkholes (Youssef et al. 2016). Moreover, infiltration of irrigation water into agricultural areas promotes dissolution of carbonates, enlargement of preexisting cavities, and sinkhole development (Urich 2002).

Figure 2 shows that earthquake epicenters and the deformation sites were found largely within areas affected by the Kahf fault system, suggesting that the presence of faults play a role in the development of deformation. The PSI results in the Wadi-As-Sirhan indicate that subsiding areas are concentrated within, but not outside of, the fault-bounded graben, an observation that suggests that the bounding faults (Figs. 3a, 10; Wadi-As-Sirhan and Al Busayta faults) were acting as subsidence barriers. Similar observations were reported from Las Vegas and surroundings (Bell et al. 2002). Our results also indicated that the observed patterns of subsidence persisted throughout the investigated period (2003–2012). The fact that high subsidence areas (bowls) within the graben do not always coincide with the areas of the highest extraction indicate that the subsidence could be affected by the distribution of low-yield, compressible fine-grained sediments. This is supported by well data from Wadi As-Sirhan, which showed the presence of significant compressible clay layers (e.g., Mira Formation; Wallace et al. 2000).

The fissures around the margins of the subsidence bowls (1, 2, 3, and 4) in Tabarjal and Busayta (Fig. 10c, Area D) were most likely caused by bending beam movements around the subsiding bowls, the regions experiencing high horizontal extension (Bell et al. 2002). Fissures also result from horizontal forces associated with compacting sediment or from horizontal seepage pressures caused by pumping; both mechanisms produce horizontal strain that is localized along planes of weakness (Helm 1994). Fissures that are located proximal to the faults could have resulted from this mechanism. An example would be the fissure proximal to the Wadi As-Sirhan fault in the lower left corner of Area E in Fig. 10c.

Our findings were consistent with those reported from aquifers worldwide (e.g., Poland 1960; Galloway et al. 1998; Hoffmann et al. 2001; Bell et al. 2002; Burbey 2002; Christiansen et al. 2007; Assumpção et al. 2010; Galloway and Burbey 2011; Avouac 2012; González et al. 2012; Martínez et al. 2013; Amos et al. 2014). The detected subsidence in these areas has been attributed to an increase in effective stress that caused compaction and land subsidence (Donaldson et al. 1995). The downward stress from the weight of the overlying rock and water was balanced by the effective stress and pore pressure. Pumping decreased pore pressure and caused an increase in the effective stress, which in turn caused compaction and land subsidence (Donaldson et al. 1995). The changes in stresses induced by groundwater extraction induce stress changes that trigger human-induced earthquakes or cause prematurely induced natural earthquakes that would have happened at a later time once natural stress is built up (Avouac 2012).

Many of the aquifers worldwide are in near-steady conditions in which the natural and anthropogenic discharge is compensated for by recharge. Examples of these conditions can be found at the Northern Great Plains Aquifer, Cambrian-Ordovician Aquifer System, and Great Artesian Basin (Richey et al. 2015). Such aquifers are expected to show near-steady GWS secular trends, near-steady secular GPS elevations, and zero radar averaged velocity (Chew and Small 2014). That is not the case with the LMAS; a fossil aquifer that was largely recharged in previous wet climatic periods, yet still currently receives modest recharge (Sultan et al. 2008). Two observations indicated that the LMAS was not at near-steady state conditions and that extraction from the LMAS was not compensated for by replenishment: (1) the depletion in TWS (− 4.1 ± 0.6 km^3^/year) and GWS (− 3.5 ± 0.6 km^3^/year) over the investigated period (Figs. 7, 8) and (2) the radar interferometric studies over Busayta region yielded subsidence rates of −4 to −15 mm/year (Fig. 10c, d).

## Conclusions

This study demonstrated that the land deformation-related features (e.g., sink holes, fissures, subsidence, and earthquakes) were caused by excessive extraction of groundwater from the fossil aquifers in central and northern Arabia. If the excessive extraction of groundwater continues, subsidence, seismicity, and structural damage to engineering structures will likewise continue, resulting in the increase in the size of existing sinkholes and fissures, as well as the formation of new ones. To minimize those hazards, the groundwater extraction has to be reduced by 3.5–4 km^3^/year, equating to the estimated annual depletion rate. Consideration should be also given to the implementation of groundwater recharge projects over the LMAS. These projects were successful in reducing water-level declines and land deformation in similar settings (e.g., Santa Clara Valley, California; Reichard and Bredehoeft 1984).

The excessive and increasing exploitation of fossil aquifers in arid lands worldwide is causing aquifer depletion and land deformation in these areas. The integrated approach presented in this study heavily relies on readily available remotely acquired data sets, and thus our procedures could potentially be used to investigate the distribution, nature, and causes of land deformation in settings similar to those of the MAS. Moreover, the adopted GRACE applications presented in this study could provide cost-effective and replicable methodologies for optimum utilization of fossil aquifers and for minimizing their associated deformational effects.

## Footnotes

1 http://www.csr.utexas.edu/grace.

2 http://www.momracors.com/portal/en/.

3 https://www.mewa.gov.sa/en/InformationCenter/OpenData/RequestOpenData/Pages/RequestOpenData.aspx.
